# Analysis of Individual and Combined Effects of Ochratoxin A and Zearalenone on HepG2 and KK-1 Cells with Mathematical Models

**DOI:** 10.3390/toxins6041177

**Published:** 2014-03-26

**Authors:** Yuzhe Li, Boyang Zhang, Xiaoyun He, Wen-Hsing Cheng, Wentao Xu, Yunbo Luo, Rui Liang, Haoshu Luo, Kunlun Huang

**Affiliations:** 1 Laboratory of Food Safety and Molecular Biology, College of Food Science and Nutritional Engineering, China Agricultural University, Beijing 100083, China; E-Mails: liyuzhe611@163.com (Y.L.); boyang0214@126.com (B.Z.); raininghe@163.com (X.H.); lyb@cau.edu.cn (Y.L.); renee.lr@qq.com (R.L.); luohaoshu@cau.edu.cn (H.L.); 2 Department of Food Science, Nutrition and Health Promotion, Mississippi State University, Mississippi State, MS 39762, USA; E-Mail: wcheng@fsnhp.msstate.edu

**Keywords:** ochratoxin A, zearalenone, combined effect, concentration addition, independent addition, risk assessment

## Abstract

Ochratoxin A (OTA) and Zearalenone (ZEA) are widespread mycotoxins that contaminate foodstuffs simultaneously, but sufficient data regarding their mixed toxicities are lacking. This study aims to analyze the style of combined effects of OTA and ZEA on cells of their target organs. For this purpose, cytotoxicity was determined in HepG2 and KK-1 cells treated with single and combined forms of OTA and ZEA. Furthermore, we have analyzed the data using two mathematical models based on the concepts of concentration addition (CA) and independent addition (IA). By analyzing data with nonlinear regression, toxins applied singly showed classic sigmoid dose-response curves in HepG2 cells whereas in KK-1 cells hormetic responses were observed. Exposure to equieffective mixtures of OTA and ZEA showed additive effects, irrespective of different nonlinear regression models used. Our results demonstrate that IA is an appropriate concept to account for mixture effects of OTA and ZEA. The results in ROS generation indicate a departure from additivity to antagonism or synergism at different concentrations, probably due to potential interaction during ROS production. This study shows that a risk assessment of mycotoxins should account for mixture effects, and prediction models are valuable tools for mixture assessment.

## 1. Introduction

Ochratoxin A (OTA) and Zearalenone (ZEA) are mycotoxins that are naturally produced by fungi, and contaminate a large variety of agricultural products. They are of major concern for animal and human health due to their toxic effects and demonstrated human exposure [1,2,3]. OTA is produced by some *Aspergillus* and *Penicillium* fungal species, and ZEA is produced by *Fusarium* species. Moreover, OTA and ZEA naturally occur in plants simultaneously [4,5] and food processing usually mixes raw materials, which makes the coexposure of mycotoxins inevitable. However, risk assessment by regulatory authorities deal with these toxicants based on the assumption that consumers are exposed to only one mycotoxin at a time. Therefore, the question is raised whether such a combined intake of OTA and ZEA would lead to a possible higher risk of adverse health effects than the intake of one of these mycotoxins alone.

OTA contaminates various foodstuffs, including cereal and grain products, coffee, beer, and wine [6]. OTA has been shown to be nephrotoxic, hepatotoxic, teratogenic and immunotoxic to humans and animals and to cause kidney and liver tumors in mice and rats [7,8]. Though its mechanisms of action are not clearly determined but apparently, OTA may disrupt phenylalanine metabolism, reduce gluconeogenesis, and induce apoptosis via protein/DNA synthesis inhibition [9]. In addition, previous work in our laboratory has shown that OTA can generate reactive oxygen species (ROS), which may explain the protein and DNA damage [10,11]. Several recent studies proposed that direct genotoxicity was the mode of action for OTA-mediated carcinogenicity [12,13,14]. However, ZEA exerts its toxic effect in a quite unique way. ZEA can bind to oestrogen receptors and compete with 17-estradiol, causing functional and morphological changes in reproductive organs [15]. The most important targets of ZEA are tissues rich in estrogen receptors, such as ovary, mammary glands, uterus, vagina, testes, epididymis, and prostate [16]. Estrogenic effects of ZEA occur at much lower concentrations than those found to elicit cell toxicity *in vitro*. Recent reports argue that the overall ZEA toxicity is not due to its estrogenicity alone [17,18]. As is demonstrated, oxidative damage is likely to be evoked as one of the main pathways of ZEN toxicity [18,19], but the exact mechanism of ZEA toxicity is not completely understood. Since OTA and ZEA act distinctively on the receptor level while at the same time, they do overlap on some mechanisms, like ROS generation, combined effect of OTA and ZEA can cause substantial and complicated changes, rendering synergistic effect, antagonistic effect or additive effect. Thus, toxicity studies on effects of OTA and ZEA mixtures are lacking.

Understanding the potential effects of a chemical mixture is a complex task requiring a sound theoretical framework that can be tested experimentally. The most well established framework to predict the combined effects of chemicals is based on the widely accepted paradigm of additivity which incorporates two basic models taking into account mode of action, these are: (1) the concentration addition (CA) model for chemicals with the same mode of action and; (2) the independent action (IA) model for chemicals with different modes of action. However, little information has been provided to assess the effects of OTA and ZEA mixtures quantitively using mathematic models.

*In vitro* assays using cells have the advantage of minimizing animal use, allowing the testing of a wide range of chemicals and concentrations [20]. OTA exerts liver toxicity depending on concentration, and human hepatoma HepG2 cells were reported to retain many of the properties of primary liver cells [21,22]. Immortalized murine ovarian granular KK-1 cells were used since reproductive organs were reported to be one of the most vulnerable targets for exposure to ZEA. The object of this study was to observe whether OTA and ZEA will affect cells from liver as well as ovarian in an additive manner. Cell viability was employed as an endpoint. Based on analyzing the dose-response relationships of the individual compounds with nonlinear regression model, the two models, CA and IA, were both applied to analyze the combined effect of OTA and ZEA. Further, as a shared mechanism of OTA and ZEA toxicity, ROS generation was assessed to explore their possible interactive behavior. We aim to develop a scientific and appropriate mathematic model to elucidate the law of combined effects of OTA and ZEA.

## 2. Results

### 2.1. Dose-Response Analysis of the Viability of HepG2 Cells Treated with OTA and ZEA Alone

**Figure 1:**
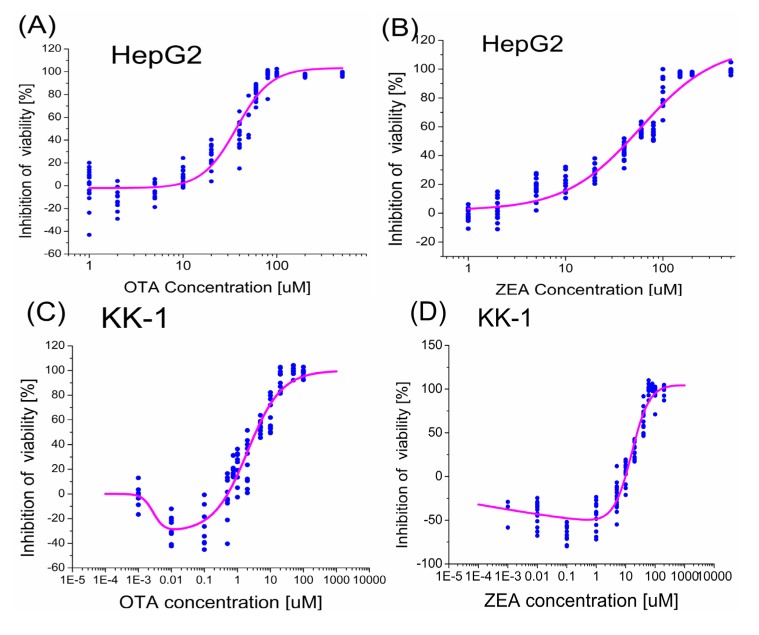
Dose-response curves for individual toxins on the inhibition of cell survival fitted by non-linear regression. The cells were exposed to mycotoxins for 24 h and the viabilitywas determined by the WST-8 assay. Inhibition of cell viability after exposure to OTA (**A**) and ZEA (**B**) of HepG2 cells, and OTA (**C**) and ZEA (**D**) of KK1 cells are shown. All experiments are repeated for at least three times in four replicates. Ochratoxin A (OTA) and Zearalenone (ZEA) inhibit the viability of HepG2 cells and KK-1 cells in a dose-dependent manner.

Reliable dose-response analyses for single substances are essential for predictions of combined toxicity. Figure 1A,B showed the experimental data and the calculated dose-response curves fitted by logistic function for single exposure of OTA and ZEA in the cell viability assay. OTA and ZEA inhibited the viability of HepG2 cells and KK-1 cells in a dose-dependent manner. The dose-response curves of OTA and ZEA are both sigmoid, but have similar effectiveness and different slopes (Figure 1). The parameters fitted from the logistic-function are summarized in Table 1, including slopes, goodness-of-fit criteria and the calculated EC_1_, EC_10_, EC_25_ and EC_50_ values. OTA was slightly more effective with an EC_50_ of 37.30 µM than ZEA with an EC_50_ of 41.28 μM, respectively. Furthermore, OTA had the higher slope of 2.65 compared with ZEA of 1.27.

### 2.2. Dose-Response Analysis of the Viability of KK-1 Cells Treated with OTA and ZEA Alone

The dose-response curves of the mycotoxins on KK-1 cells are not simply sigmoid, but showed hormetic shape, which is characterized by a low-dose stimulation and a high-dose inhibition. The toxicity data in the cell viability assay were fitted using the hormetic model (Function 3), and the dose-response curves are shown in Figure 1C and Figure 1D. The fitted parameters and calculated toxicity values are listed in Table 2. OTA was more potent, with EC_50_ up to 7-fold lower than that calculated for ZEA. However, ZEA was found to have a more significant stimulatory effect on the viability of KK-1 cells, with the stimulation peak value being 60% compared with that of OTA at around 30% (Figure 1C,D).

**Table 1:** Parameters of the dose-response curves fitting with logistic function of the individual OTA and ZEA effects on HepG2 cells and their concentrations in equieffective mixtures of two compounds.

Mycotoxins	Dose-response models			Binary mixture ratios (μM)			
	Slope ^a^	R^2 b^	Chi^2^/DoF ^c^	EC_1_	EC_10_	EC_25_	EC_50_
OTA	2.65 ± 0.37	0.9384	120.52	6.61	16.30	24.66	37.30
ZEA	1.27 ± 0.13	0.9248	96.04	1.12	7.36	17.44	41.28

Notes: ^a^ Slope parameter of the logistic function, expressed with 95% confidence intervals; ^b,c^ Goodness of fit criteria of the logistic function.

**Table 2:** Parameters of the dose-response curves fitting with hormetic model of the individual OTA and ZEA effects on KK-1 cells and their concentrations in equieffective mixtures of two compounds.

Mycotoxins	Dose-response models						Binary mixture ratios (µM)			
	P_1 _^a^	P_2 _^b^	P_3 _^c^	P_4 _^d^	R^2 e^	Chi^2^/DoF ^f^	EC_1_	EC_10_	EC_25_	EC_50_
OTA	0.00281 ± 0.0049	2.60 ± 4.07	2.04 ± 0.29	0.84 ± 0.10	0.9226	170.32	0.53	0.80	1.44	3.61
ZEA	0.00010 ± 0.0005	0.15 ± 0.11	15.97 ± 2.42	1.41 ± 0.14	0.9528	193.41	9.98	11.84	15.50	24.40

Notes: ^a,b,c,d^ Parameter for hormesis model, expressed with 95% confidence intervals; ^e,f^ Goodness of fit criteria of the function.

### 2.3. Models Analysis of the Combined Effects of OTA and ZEA on Cell Viability

**Figure 2:**
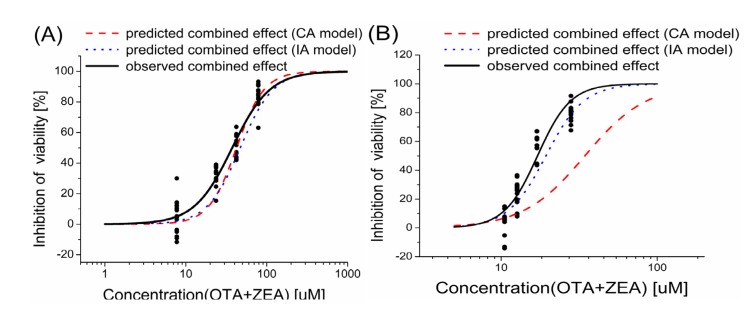
Comparison of observed and predicted cell death after exposure to mixtures of OTA and ZEA. Components are mixed equieffectively in the ratio of their individual EC_1_, EC_10_, EC_25_ and EC_50_ values. EC_1_, EC_10_, EC_25_ and EC_50_ stand for concentrations causing 1%, 10%, 25% and 50% of cytotoxicity. The predicted dose-response curves of the mixture were fitted with the data calculated from CA and IA models. All curves were fitted by the logistic function. (**A**) HepG2 cells; (**B**) KK-1 cells.

Because of the difference in potencies observed for the tested compounds in this study, equipotent concentrations were used when designing the mixtures in order to provide similar contribution from each compound to the combined effect. In this study, the mixture of OTA and ZEA was designed in the equivalent effect of their individual EC_1_, EC_10_, EC_25_ and EC_50_ values (Table 1 and Table 2), which were derived from their dose-response curves. A dose-dependent inhibition of cell viability was demonstrated after treatment of the cells with the combined mycotoxins. To evaluate what kind of combined effect was provoked by the mixture, the observed results caused by the mixture are compared with the modeled effects based on the concept of CA and IA (Figure 2). The comparison between the predicted concentration-response curves fitted based on the CA and IA models and the observed curves summarized with experimental data was shown in the Figure 2 and Table 3.

In HepG2 cells (Figure 2A and Table 3), the results of cell viability demonstrate that both prediction models aligned well with the observed effect of the mixture, especially the IA prediction.

In the KK-1 cells (Figure 2B), the IA model described the mixture data very well, as all MDRs predicted by IA were within a factor of 2 (Table 3) and were consistent with the assumption of additivity. However, the CA model predicted effect deviated markedly from the observed effect. 

**Table 3:** Calculated model deviation ratios (MDRs) at different observed effect levels (% effect) for the mixture.

Inhibition level	HepG2				KK-1		
	Observed inhibition effect	MDR CA	MDR IA	Observed inhibition effect		MDR CA	MDR IA
1%	3.85	2.15	0.52	2.33		2.36	0.85
10%	33.05	0.81	0.57	23.30		0.39	0.82
25%	50.17	0.78	0.87	56.31		0.35	0.78
50%	82.62	1.12	0.91	79.72		0.48	0.94

Note: Dark gray color indicates effect levels where the CA or IA prediction deviates by more than a factor of two (MDR > 2 or MDR < 0.5) from the experimental dose-response curves.

### 2.4. ROS Generation Induced by OTA and ZEA Alone and in Combination in Two Cell Models

Upon interaction with ROS, the fluorochrome DCFH-DA is converted to the fluorescent DCF. We treated HepG2 cells and KK-1 cells for 24 h with OTA and ZEA of their individual EC_1_, EC_10_, EC_25_ and EC_50_ concentrations obtained in the viability assay and their mixtures combined at same individual effect levels.

**Figure 3:**
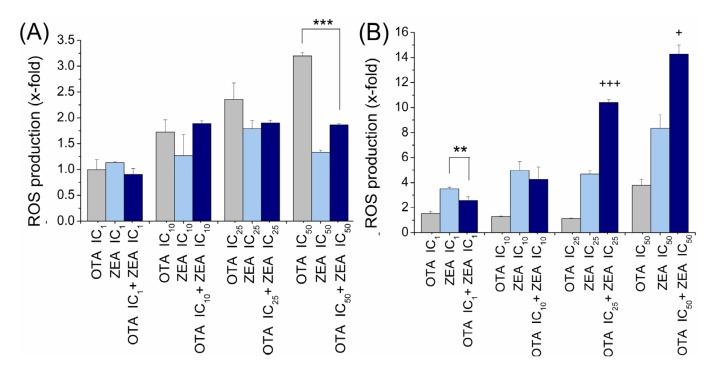
Individual and combined effects of OTA and ZEA on the intracellular ROS production. Total intracellular ROS were measured using DCFH-DA by flow cytometry in HepG2 cells (**A**) and KK-1 cells (**B**) exposed to OTA, ZEA or mixture (OTA + ZEA) for 24 h. The relative DCF-fluorescence intensity (fold) in treated cells is shown. Bars represent the mean ± SD. “*” indicates that the value of the mixture group is significantly lower than the individual mycotoxin group, showing antagonistic. “+” indicates that the value of the mixture group is significantly higher than the value of adding the two individual groups’ value together, showing synergistic. (*, +, *p* < 0.05; **, ++, p < 0.01: ***, +++, p < 0.001).

For HepG2 cells (Figure 3A), there was a dose-dependent induction of ROS formation by OTA, but not ZEA treatment. When the cells were treated with both OTA and ZEA, there was no significant additive effect. What is interesting is that the exacerbation of ROS triggered by OTA can be effectively inhibited by the addition of ZEA on the upper dose level of EC_50_.

In KK-1 cells (Figure 3B), we observed an insignificant ROS induction through OTA exposure except a sudden increase at the EC_50_ cell viability effect level. ZEA treated cells suffered a dose-dependent increase in ROS formation. In the ROS assay for mixture we found an interesting result when compared with the toxins used alone, that at lower concentrations, which induced the individual effect of death rate under 10%, the combined effect was characterized by possible antagonism, whereas at higher concentrations (individual EC_25_ and EC_50_), significant synergistic effect was observed.

These two cell models showed similar patterns of ROS induction when exposed to the tested mycotoxins. OTA and ZEA, single and in combination, increased ROS levels in both the two cell models. Furthermore, the two cell lines both exhibit non-additive or even antagonism effects when exposed to mycotoxin mixtures at low concentrations. However, the two cell models exhibited quite different patterns of combined effects, synergism or antagonism respectively, in upper concentrations (individual EC_25_ and EC_50_).

## 3. Discussion

Mycotoxins contaminate foods and feeds worldwide. They coexist in plants naturally and the manufacturing process usually mixes together different raw materials. Therefore, it is necessary to take their combined toxicity into account. Surprisingly, most toxicity studies have been carried out by considering only conditions of single mycotoxin exposure. In the present study, the combined toxicity of the major mycotoxin OTA and ZEA has been investigated. Since OTA is hepatotoxic and ZEA mainly harms reproductive organs, hepatocyte and ovarian granular cells were selected to explore the combined effect of the two mycotoxins. These results showed that, although OTA and ZEA act though distinct mechanisms, co-treatment with both OTA and ZEA additively affects cell viability, suggesting the need to consider the combined effect of mycotoxins for risk assessment.

To analyze the predictability of the combined effects of OTA and ZEA, it is necessary to examine the individual toxic effect of each mycotoxin. For the HepG2 cell line, both the two mycotoxins elicit a dose-dependent inhibition of cell viability when tested alone, showing typical sigmoid curves. Their toxic potency is similar, as reflected by the EC_50_ values. Using the same cell model, recent studies have shown identical sigmoid-shaped response curves but higher EC_50_ for inhibitory effects of OTA and ZEA on cell viability using the fluorescein diacetate assay [23]. Differences in the assessments used may have accounted for the observed differences in cytotoxicity. As for the KK-1 cell line, the two mycotoxins show hormetic biphasic but not monotonic sigmoid dose-dependent curves, exhibiting a stimulatory effect at low concentrations but an inhibitory effect at high concentrations. The stimulation effect is in agreement with the generally reported stimulating amplitude of about 30%–60% [24]. Similarly, Zhu *et al*. show that cell viability of primary porcine granulosa cells is decreased by ZEA at concentrations greater than 60 μM [25], and other studies have demonstrated that low concentrations (<0.1 mM) of ZEA stimulate the proliferation of equine granulosa cells [26]. However, the hormetic phenomenon of OTA has not been reported. In recent years, an increasing body of evidence indicates that hormesis is a universal cell response. Calabrese [27] provided substantial documentation suggests that hormetic dose-responses occur in many types of tumor cells, independent of organ types. Temporal factor is a complication that affects the ability to observe hormesis in laboratory studies [28], which may explain the response of HepG2 fitting a threshold model rather than hormesis. Therefore, these two models applied both provide statistically valid prediction of cytotoxic effects of mycotoxins. The WST-8 assay employed in the current study provides reproducible data with relatively few variations and the parameters are determined based on well fitted dose-response curves, which makes the assessment more credible and precise.

In order to determine if OTA and ZEA interact, we have analyzed the binary mixtures at equi-effective concentrations of EC_1_, EC_10_, EC_25_ and EC_50_, and observed a combined effect of these two mycotoxins. Impairment of cell viability after mixture exposure is stronger than that caused by the toxins applied alone, irrespective of cell types. Previous data on combined toxic effects of OTA and ZEA are generally limited, but it has been shown that either the mycotoxins do not share cell pathways in their toxic action, or if they share some step, it is expected that they would at least have additive effects [5]. A study demonstrated synergistic effects of aflatoxin B1 and fumonisin B1 on cell proliferation in rat spleen mononuclear cells [29]. Similarly, combined effects of binary and ternary mixtures of ochratoxins A and B, citrinin and patulin have been reported on the porcine renal cell line LLC-PK1 using a MTT cytotoxicity assay [30]. In a human system, Ficheux *et al*. [31] observed mainly additive myelotoxic effects when testing several binary fusariotoxin mixtures. These indicate that combined effects of mycotoxin mixtures are generally observable. However, synergistic and additive effect is not always the case; a study assessing the combined cytotoxicity of three fusarium mycotoxins found all mycotoxin combinations show antagonistic interactions [32]. Regardless of the diverse patterns of joint effects, few studies published so far have provided the mathematic model applied for the estimation of the individual dose-response functions or the estimated parameter values. In our study, dose-response relationships are determined not only for the individual substances but also for the combined effect. Furthermore, we have formulated a model describing the expected combined effect.

The two prediction models applied in the present study test two classic concepts of additivity, concentration addition (CA) and independent action (IA). Both concepts allow for calculating the expected combined toxicity, based on the toxicities of the individual compounds and their concentrations in the mixture. The choice of reference model, CA or IA, is theoretically based on the molecular target of a compound, or more broadly defined, its mode of action [33]. Concentration addition assumes that the compounds bind to the same receptor. Substances have a similar mechanism of action, and they do neither interact on a physicochemical level nor in their toxicokinetics and toxicodynamics [34]. Independent action assumes that the mixture components act on different subsystems (tissues, cells, molecular receptors) of an exposed organism and that impaired sub-systems affect the end point under observation independently of each other [35].

Data from the current study demonstrate that the IA concept accurately predicts the combined effect of OTA and ZEA in both two cell models. The CA concept also succeeds in predicting the joint effects in the HepG2 cell but fails in the KK-1 cell model. The agreement between experimental data and predictions are assessed with MDR. The majority of the MDRs for IA of the binary mixture are within a factor of 2 (0.5 ≤ MDR ≤ 2), but some of the MDRs for CA are beyond this cutoff point (Table 3). Overall, the combined effect of the mycotoxin mixture studied here is more precisely predicted by the IA concept than by the CA concept. Therefore, it seems plausible to suggest that the tested mycotoxins, OTA and ZEA, act independent of each other. This agrees with previous findings of the toxic mechanism of OTA and ZEA on cells, in which the primary biochemical lesions leading to toxic cell injury of the two mycotoxins are different. OTA disrupts phenylalanine metabolism as an early cellular event [5] when the interactions are thought to be exerted through specific binding. Because OTA is a structural analogue of phenylalanine, it in principle can act on all metabolic systems involving phenylalanine [36,37]. Consistent with this, competitive inhibition by OTA *versus* phenylalanine of the Phe-tRNA synthase is the primary cause of OTA toxicity [38]. Further, a recent study confirms that OTA does not interact with steroid receptors [39]. However, ZEA binds to and activates estrogen receptors, because the structure of ZEA is similar to that of 17β-estradiol [16]. The strong estrogenic activity of ZEA accounts for several physiological alterations of the reproductive tract in laboratory and farm animals [40]. Since the CA and IA concepts are distinguished by whether they act on the same molecular receptor or not, and OTA and ZEA do interact differently with endogenous molecules, the joint results in accordance with IA concept prediction can thus be reasonably explained.

However, chemicals usually have unknown or multiple modes of action. As in the case of OTA and ZEA, although they differ in receptor binding characteristics, they share some common mechanisms of action, such as induction of DNA adducts [41,42,43] and endocrine disruption [39,44]. Furthermore, they both induce ROS generation and trigger a p53-dependent apoptotic pathway [23] in HepG2 cells. These common mechanisms may partly explain the principle of CA on the accurate prediction of the combined toxicity in HepG2 cells.

The present work provides further information on the combined effect of OTA and ZEA on ROS induction. ROS production is a shared mechanism of the two chemicals and thus may be related with their cytotoxicities. ROS production is assessed in cells incubated for 24 h with OTA and ZEA, alone or in combination, at their respective concentrations of EC_1_, EC_10_, EC_25_ and EC_50_ of cell viability determined by WST-8 assay. However, ROS may not be directly linked to cell survival when both OTA and ZEA are present. Indeed, the default models of CA or IA fail to describe the combined toxicity of OTA and ZEA. Especially in their upper concentrations (combined at their individual EC_25_ and EC_50_), the two cell models do not exhibit perfect additive effect. Even in the same cell model, the combined effect differs depending on the actual dose of the mixture. At low doses an apparent antagonism is observed, whereas synergy is observed at high doses in KK-1 cells (Figure 3). This would imply that, as an overlapped metabolic response, ROS stimulation may occur as a result of mycotoxins interaction. Similar result has been reported on cytotoxicity of metal mixtures, which can be synergistic, antagonistic, or additive depending on the doses or ratios of the metals in a mixture [45,46]. And they attribute the discrepancies between observed and predicted responses to compounds interaction. Altogether, we propose that ROS may play a dual role, by acting as toxic bioproducts that alter the cellular function and viability, and as key regulators of cellular signaling pathways. Both these actions may be related to the OTA and ZEA individual or combined toxicities [29]. To the best of our knowledge, direct oxidative interaction between OTA and ZEA has not been reported. The data presented in the current study suggest that oxidative damage is not the only cause of OTA and ZEA toxicity.

There’s a contradiction between results of the cell viability assay and ROS generation assay. While the combined cytotoxicity of OTA and ZEA is additive and can be accurately predicted by the additive model, the ROS assay suggests antagonistic or synergistic effect. Similarly, the combined effects of AFB1 and OTA are additive based on cytotoxic assays but are antagonistic based on genotoxic studies [9]. We then hypothesis speculatively that, the overall joint cytotoxicity maybe follow additive rules, but regarding different toxic mechanism and specific endpoints, they may show diverse combined effects. The mechanism of mycotoxins’ combined effect is complicated, involving chemicals’ interactions with cell receptors and enzymes, and can not be elucidated easily. Though estrogenic effects of ZEA occur at much lower concentrations than those found to elicit cell toxicity *in vitro*, there is a higher probability of being exposed to low doses of several mycotoxins at the same time throughout life than to a high level of only one mycotoxin. The current study provides a simple and applicable model to assess the overall combined toxicity of mycotoxins in the context of food safety. An important task will be to investigate whether our findings hold true at the molecular level, e.g., biological receptors, at much lower concentrations. At present, the available database concerning the possible effects of combined exposure of mycotoxins is insufficient for establishing the consequences of mycotoxin interactions. Thus, the combined toxicological effect of mycotoxins should be determined or revalidated.

## 4. Experimental Section

### 4.1. Chemicals

ZEA was obtained from Sigma-Aldrich (St. Louis, MO, USA), and OTA was obtained from Fermentek (Jerusalem, Israel). Fetal bovine serum (FBS) was obtained from HyClone (Logan, UT, USA). The Cell Counting Kit-8 and fluorescence probe DCFH-DA were obtained from Beyotime (Jiangsu, China).

### 4.2. Cell Cultures and Treatment

HepG2 and KK-1 cells were cultured in high-glucose Dulbecco’s minimal essential medium supplemented with 10% fetal bovine serum (HyClone, Logan, UT, USA), 100 U/mL penicillin, 100 μg/mL streptomycin and 250 ng/mL amphotericin B (Macgene, Beijing, China) at 37 °C in a humidified 5% CO_2_ incubator (Sanyo, Tokyo, Japan).

OTA and ZEA were diluted with serum-free medium. After seeding the cells on plates or in single wells we exposed them with OTA, ZEA or their mixture for 24 h. The cells were then dispersed with a solution of 0.25% trypsin (w/v) and 0.52 mM EDTA (Macgene, Beijing, China).

### 4.3. Cell Viability Assay

Cell viability was determined using Cell Counting Kit-8 according to manufacturer’s instructions. In brief, 1 × 10^4^ cells/well were seeded in a 96-well flat-bottomed plate, grown at 37 °C for 24 h, treated with different concentrations of OTA and ZEA, alone or combined, for 24 h, and washed once with phosphate-buffered saline (PBS). Subsequently, 10 μL of water-soluble tetrazolium salt (WST-8) dye and 100 μL of PBS were added to each well, and cells were incubated at 37 °C for 1 h. Finally, dye absorbance of reduced WST-8 was determined at 450 nm using a plate reader (Thermo, Waltham, MA, USA) [47]. The viability was calculated using Equation (1). This experiment was repeated at least three times in four replicates for OTA and ZEA alone or in combination.


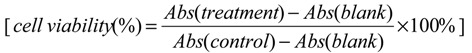
(1)

### 4.4. ROS Production Assay

The cells were cultured in a 6-well plate at a cell density of 2 × 10^5^ cells/well. After exposure to OTA, ZEA or their mixture for 24 h, the cells were then loaded with 10 μΜ DCFH-DA (Beyotime, Shanghai, China) and incubated at 37 °C for 20 min. The cells were washed twice with serum-free medium and trypsinized. Then, fluorescence intensity was determined by FACSCalibur (BD Biosciences, San Jose, CA, USA). At least 1 × 10^4^ cells in gate were collected for flow cytometry analysis.

### 4.5. Statistical Analysis

The data were expressed as the means ± SD. The experiments were repeated at least twice, and each experiment included at least triplicate treatments. The data from the different treatments were subjected to an analysis of variance (ANOVA), and the comparisons of the means were performed using Duncan’s multiple range test. All of the statistical analyses were performed using SPSS 13.0. Differences with P values less than 0.05 were considered significant.

#### 4.5.1. Model for Single Effect

In order to accurately predict the interaction between OTA and ZEA, it is necessary to determine the effect of individual mycotoxin on the viability of cells in advance. The single toxin concentration ranges were chosen to cover as much of the dose-response curve as possible, preferably from 1% to 95% predicted effect. Each data set comprised at least 12 different concentrations. The dose-response relationships of the individual substances were biometrically modeled using the mathematical software Origin 7.5 (Origin-Lab, North Hampton, MA, USA). The parameters of the models for single mycotoxin were then obtained by a non-weighted nonlinear regression analysis. Goodness-of-fit criteria included the 95% confidence interval (CI) of the fitted parameters, the coefficient of determination (R^2^), the Chi-square per degree of freedom (Chi^2^/DoF). Calculation of concentration causing certain rate of cytotoxicity, 1%, 10%, 25% and 50% effect concentrations (EC_1_, EC_10_, EC_25_ and EC_50_), was also performed with Origin 7.5.

For HepG2 cells, the logistic-model was fitted to the experimental data set of effects of the two mycotoxins. The logistic equation is mathematically analogous to the Hill equation usually used for ligand binding and enzyme kinetics [48]. The two parameter logistic-function is shown in Equation (2), where *E* = effect in %; *p* = slope; *EC* = effect concentration; *x* = concentration (µM).


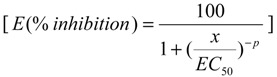
(2)

For KK-1 cells, the two mycotoxins inhibited cell viability at high concentration levels, but they presented apparent stimulatory effects at low concentrations. Thus, a hormetic model [49] was applied to predict the dose-response curve precisely. The hormetic dose-response curve was divided into two parts, and each theoretical curve was fitted using a logistic model. By adding up the two logistic models, a new model that can fit the real hormetic dose-response was obtained. The new model is considered to be bi-logistic because it was obtained via the algebraic summation of two logistic functions representing the stimulation and the inhibition. Equation (3) was used where *a* and *b* are the first median and slope in the low-dose region, respectively; *p* and *q* are the second median and slope in the high-dose region, respectively; *m* is the bottom; and *x* is the concentration of the mycotoxin. More details about the new model are provided in the result section.


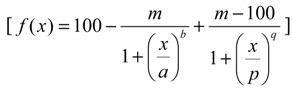
(3)

#### 4.5.2. Design Strategy for Test Mixture

The two mycotoxins were mixed at equieffective concentrations on basis of their ability to inhibit cell viability; this means that the concentrations used for the mixture would result in the same effect when applied alone. The binary mixture experiments were conducted by mixing effect concentrations of the mycotoxins for their individual effect levels of EC_1_, EC_10_, EC_25_ and EC_50_, respectively. Table 1 provides data derived from their dose-response curves and the respective concentration values used in the mixture study.

#### 4.5.3. Mathematical Model for Mixture Effect

The main hypothesis when testing effects of mixtures is that the mixed chemicals exert an additive effect. The models most acknowledged by authorities in this field are the model of concentration addition (CA) and the model of independent action (IA) [50,51,52]. The effects of the mixture were predicted with the two models based on dose-response regression curves of the individual mycotoxins.

In the CA model, it is assumed that the compounds act by similar mode of action and can replace each other [50]. The model of CA states that:

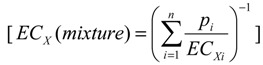
(4)
where, *EC_x_(mixture)* is the predicted total concentration of the mixture that produces X% effect; *p_i_* is the relative fraction of component *i* in the mixture and *EC_Xi_* is the concentration of substance *i* provoking a certain effect when applied alone. 

The IA model assumes that the compounds act dissimilarly. The basic version of independent action has been formulated under the assumption that the susceptibilities of the individuals of an at-risk-population to different dissimilarly acting mixture compounds are not correlated with each other [35]. For a mixture of i components this can be defined as:

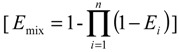
(5)
where, *E_mix_* is the effect of the mixture of n compounds; and *E_i_* is the effect of substance *i* when applied singly.

The experimentally observed combined effects were then compared with the predictions. The accuracy of the prediction models was assessed by comparing the model-predicted combined effect with the experimentally derived dose-response curves. In addition, the model deviation ratio (MDR) was calculated as the ratio between the predicted effect concentration and the experimental effect concentration at the different effects levels. A MDR of greater than one indicated that the model under-predicted toxicity, whereas a value of less than one indicated that the model over-predicted toxicity. A MDR within a factor of 2 from the model prediction (0.5 ≤ MDR ≤ 2) indicated that the mixture was most likely to follow the principle of addition as this ratio is within the expected inter-laboratory/inter-experiment deviation for most species [52].

#### 4.5.4. Analysis of Single and Combined Effect on ROS Generation

It is hard to set a 100% rate of cell’s ROS induction, so we didn’t use the addition models to predict and evaluate their combined effect on induction of ROS formation. But we can conduct from thefunctions of additive model that, if the mixture exert additive effects on cells, it will follow the rules showed below.

[*E*_1_*orE*_2_ < *E*_1+2_ < *E*_1_ + *E*_2_] (6)

The expected additive effect of two substances (*E*_1*+*2_) is confined to the region between the value of adding the effect of single substances (*E*_1_ *+ E*_2_) and the single effect with the higher value. Thus, if the observed combination value exceeds the value of adding the effect of single substances (*E*_1_ *+ E*_2_), it indicates synergism. And, if the observed combined value is less than either one of the single substances, it shows antagonism. All of the statistical analyses were performed using SPSS 13.0. Differences with P values less than 0.05 were considered significant.

## 5. Conclusions

In summary, by using mathematical models to predict mixture toxicity, our results indicate that additivity is an important property of OTA and ZEA, and should be considered in food safety assessment. We found that the combined toxicity can be modeled by the CA and IA concepts, and the IA model provides quite accurate results. We suggest that both models can be valuable tools in assessing the toxicity of complex natural mixtures of mycotoxins.
